# Robust brain tumor classification by fusion of deep learning and channel-wise attention mode approach

**DOI:** 10.1186/s12880-024-01323-3

**Published:** 2024-06-17

**Authors:** Balamurugan A.G, Saravanan Srinivasan, Preethi D, Monica P, Sandeep Kumar Mathivanan, Mohd Asif Shah

**Affiliations:** 1 Department of Computer Science and Engineering, Vel Tech Rangarajan Dr. Sagunthala R&D Institute of Science and Technology, Chennai, 600062 India; 2 Department of Computer Science and Engineering, Faculty of Engineering and Technology, SRM Institute of Science and Technology, Ramapuram , Chennai, India; 3 School of Electrical and Electronics Engineering, VIT Bhopal University, Bhopal, Indore Highway, Kothrikalan, Sehore, Madhya Pradesh 466114 India; 4 School of Computer Science and Engineering, Galgotias University, Greater Noida, 203201 India; 5 Department of Economics, Kardan University, Parwan-e-Du, Kabul, 1001 Afghanistan; 6 Division of Research and Development, Lovely Professional University, Phagwara, Punjab 144001 India

**Keywords:** Brain tumor, Deep learning, ResNet101, CWAM, Attention mechanism

## Abstract

Diagnosing brain tumors is a complex and time-consuming process that relies heavily on radiologists’ expertise and interpretive skills. However, the advent of deep learning methodologies has revolutionized the field, offering more accurate and efficient assessments. Attention-based models have emerged as promising tools, focusing on salient features within complex medical imaging data. However, the precise impact of different attention mechanisms, such as channel-wise, spatial, or combined attention within the Channel-wise Attention Mode (CWAM), for brain tumor classification remains relatively unexplored. This study aims to address this gap by leveraging the power of ResNet101 coupled with CWAM (ResNet101-CWAM) for brain tumor classification. The results show that ResNet101-CWAM surpassed conventional deep learning classification methods like ConvNet, achieving exceptional performance metrics of 99.83% accuracy, 99.21% recall, 99.01% precision, 99.27% F1-score and 99.16% AUC on the same dataset. This enhanced capability holds significant implications for clinical decision-making, as accurate and efficient brain tumor classification is crucial for guiding treatment strategies and improving patient outcomes. Integrating ResNet101-CWAM into existing brain classification software platforms is a crucial step towards enhancing diagnostic accuracy and streamlining clinical workflows for physicians.

## Introduction

The brain, which serves as the central command centre of the body, controls bodily functions and plays a vital role in maintaining general health. Brain tumours and other anomalies can present substantial hazards. Malignant tumours, which are characterised by the rapid and aggressive proliferation of cells, provide significant challenges in terms of management due to their fast growth. Conversely, benign tumours, although less menacing, can nonetheless lead to difficulties [1]. Accurate diagnosis and treatment planning require a thorough understanding of the distinction between malignant and benign tumours. Progress in medical technology and research is constantly enhancing the effectiveness of therapies for brain tumours, leading to better results for patients [2]. The World Health Organisation (WHO) has devised a classification system for brain tumours, categorising them into four groups. Tumours classified as Grade I and II are considered lower-grade and have a more favourable prognosis. Tumours classified as Grade III and IV are characterised by a more severe nature, displaying aggressive behaviour and resulting in poorer outcomes [3]. Comprehending these grades is essential for clinicians to customise treatment methods and offer precise prognosis information. This technique enables healthcare practitioners to categorise individuals according to the severity of their tumours, so improving the effectiveness of treatment and the outcomes for patients. Brain tumours present a substantial risk to life, and precise diagnosis is essential for successful treatment. Magnetic Resonance Imaging (MRI) and Computed Tomography (CT) scans, in conjunction with biopsy procedures and pathological examinations, are employed to validate diagnosis [4]. MRI is favoured since it is non-invasive. Nevertheless, manual examination poses difficulties and inaccuracies. Computer-Aided Diagnosis (CAD) approaches have transformed the discipline by employing artificial intelligence and machine learning. These algorithms aid neuro-oncologists in the identification, classification, and grading of tumours, improving diagnostic precision and optimising workflows [5]. This method enhances patient outcomes in the intricate realm of brain tumour identification and therapy. The application of deep learning techniques has greatly enhanced computer-assisted medical diagnosis, specifically in the detection and classification of brain tumours. Transfer learning, a branch of artificial intelligence, has demonstrated promise in tasks such as visual categorization, object identification, and image classification [6]. Neuro-oncology researchers have employed pre-trained networks to extract characteristics from brain MRI scans, resulting in a remarkable accuracy rate of 98.58%. Convolutional neural network architectures such as AlexNet and Shuffle-Net have been assessed for their ability to extract features and classify data [7]. Convolutional neural networks (CNNs) are crucial in the prediction of brain tumours, as they extract diverse features using convolution and pooling layers. Nevertheless, there is a limited availability of attention-based models for the categorization of brain tumours. The predominant approach in current models is the utilisation of Convolutional Neural Networks (CNNs) and transfer learning approaches [8]. Several research have employed 3D-CNNs with innovative network structures for the categorization of multi-channel data, resulting in an accuracy rate of 89.9%. Prior research has concentrated on dividing brain tumours in MRI imaging by utilising fully convolutional neural networks [9]. Recent advancements have combined traditional architectural elements with CNN principles, such as correlation learning mechanisms (CLM) for deep neural network architectures in CT brain tumor detection, achieving an accuracy rate of 96% [10]. Research in brain tumor image classification has also explored the effectiveness of architectures like AlexNet, GoogLeNet, and ResNet50. The study presents two deep learning models for brain tumor classification, ResNet50 and VGG16. ResNet50 has the highest accuracy rate at 85.71%, indicating its potential for brain tumor classification [11]. The models were trained on a comprehensive dataset of 3,064 and 152 MRI images, sourced from publicly available datasets. The VGG16 architecture achieved classification accuracies of approximately 97.8% and 100% for binary and multiclass brain tumor detection, respectively [12].

Nevertheless, additional enhancements are required. The objective of the work is to incorporate an attention mechanism into the brain tumour classification task, since it has been demonstrated to improve the detection of important characteristics in intricate datasets. This integration has the potential to enhance accuracy rates and minimise misclassifications, resulting in more precise diagnoses and better patient outcomes [13]. The work offers a potential path for improving and perfecting algorithms used to classify brain tumours. The author employed the recurrent attention mechanism (RAM) model and channel attention mechanism to enhance the classification accuracy of biomedical images. According to [14], the RAM model demonstrated superior performance compared to typical CNNs when dealing with difficulties in imaging data.

The channel attention mechanism, which focuses on brain tissue spatial distribution, was also integrated into the classification process. This approach improved the accuracy of identifying and categorizing brain tumors based on their spatial characteristics. These techniques offer promising avenues for medical image analysis, leading to more accurate diagnoses and improved patient outcomes [15]. This proposed study presents a novel approach to brain tumor classification by combining deep learning techniques with channel-wise attention mechanisms. The study focuses on enhancing the accuracy and efficiency of brain tumor classification, crucial for effective diagnosis and treatment planning. Through the fusion of deep learning models and attention mechanisms, the proposed method aims to improve feature extraction and classification accuracy. The paper outlines the methodology, experimental results, and discusses the implications of the findings for future research and clinical applications. Overall, the study contributes to advancing the field of medical image analysis and underscores the importance of integrating innovative techniques for improved brain tumor classification. The research contribution of this study is as follows,

- Utilization of Channel-wise Attention Mechanism: The proposed approach leverages the Channel-wise Attention mechanism to accurately classify different types of MRI images of the brain, including glioma, meningioma, no tumor, and pituitary classes. This mechanism allows the model to focus on relevant features within the images, thereby improving classification accuracy.
- Effective Data Preprocessing: The study emphasizes the importance of effective data preprocessing techniques, which likely contributed to the high accuracy of the proposed method. Proper preprocessing helps ensure that the input data is clean, standardized, and well-suited for training deep learning models.
- Integration into Clinical Decision-Making: Given the impressive performance of the proposed method, the authors advocate for its integration into software platforms used by physicians. This integration has the potential to enhance clinical decision-making and ultimately improve patient care by providing more accurate and efficient diagnosis of brain tumors.
- Future Research Directions: The study outlines future research directions, including the utilization of additional brain tumor datasets and exploration of different deep learning techniques to further enhance brain tumor diagnosis. This highlights the researchers’ commitment to ongoing improvement and innovation in the field.
- Identification of Computational Complexity: The study also identifies the computational complexity associated with the proposed model, particularly due to the addition of CWAM attention modules to the ResNet101 architecture. Understanding and acknowledging these limitations are essential for guiding future research efforts and optimizing model development processes.

The structure of this paper is as follows: Chap. 2 discusses about the recent state-of-the-art methods and its outcomes. Chapter 3 provides details about the dataset utilized in this study and outlines the complete structure of the proposed classification algorithm. Chapter 4 presents the experimental results obtained through the methodology. Chapter 5 discusses the conclusions drawn from the study and outlines avenues for future research concerning the proposed model.

## Related work

Palash Ghosal et al. (2019) [16] brain tumors pose a significant threat to life and socio-economic consequences. Accurate diagnosis using MRI data is crucial for radiologists and minimizing risks. This research introduces an automated tool for classifying brain tumors using a Squeeze and Excitation ResNet model based on ConvNet. Preprocessing techniques like zero-centering and intensity normalization are used, resulting in an accuracy rate of 93.83%. This approach shows promising advancements in sensitivity and specificity compared to current methods. Wenna Chen et al. (2024) [17] brain tumor classification is crucial for physicians to develop tailored treatment plans and save lives. An innovative approach called deep feature fusion uses convolutional neural networks to enhance accuracy and reliability. Pre-trained models are standardized, fine-tuned, and combined to classify tumors. Experimental results show that combining ResNet101 and DenseNet121 features achieves classification accuracies of 99.18% and 97.24% on Figshare and Kaggle datasets, respectively. Muhannad Faleh Alanazi et al. (2022) [18] presents a transfer learning model for early identification of brain tumors using magnetic resonance imaging (MRI). The model uses convolutional neural network (CNN) models to assess their efficacy with MRI images. A 22-layer binary classification CNN model is then fine-tuned using transfer learning to categorize brain MRI images into tumor subclasses. The model achieves an impressive accuracy of 95.75% when tested on the same imaging machine. It also shows a high accuracy of 96.89% on an unseen brain MRI dataset, indicating its potential for real-time clinical use.

Hanan Aljuaid et al. (2022) [19] breast cancer is a global issue, with increasing frequency due to insufficient awareness and delayed diagnoses. Convolutional neural networks can expedite cancer detection and classification, aiding less experienced medical practitioners. The proposed methodology achieves top-tier accuracy rates in binary and multi-class classification, with ResNet, InceptionV3Net, and ShuffleNet achieving 99.7%, 97.66%, and 96.94% respectively. Nazik Alturki et al. (2023) [20] brain tumors are among the top ten deadliest illnesses, and early detection is crucial for successful treatment. A study uses a voting classifier combining logistic regression and stochastic gradient descent to distinguish between cases with tumors and those without. Deep convolutional features from primary and secondary tumor attributes enhance precision. The voting classifier achieves an accuracy of 99.9%, outperforming cutting-edge methods.

Ginni Arora et al. (2022) [21] this study focuses on evaluating the effectiveness of deep learning networks in categorizing skin lesion images. The research uses a dataset of approximately 10,154 images from ISIC 2018, and the results show that DenseNet201 achieves the highest accuracy of 0.825, improving skin lesion classification across multiple diseases. The study contributes to the development of an efficient automated classification model for multiple skin lesions by presenting various parameters and their accuracy. Jun Cheng et al. (2015) [22] this study focuses on classifying three types of brain tumors in T1-weighted contrast-enhanced MRI (CE-MRI) images using Spatial Pyramid Matching (SPM). The method uses an augmented tumor region generated through image dilation as the ROI, which is then partitioned into fine ring-form subregions. The efficacy of the approach is evaluated using three feature extraction methods: intensity histogram, gray level co-occurrence matrix (GLCM), and bag-of-words (BoW) model. The results show substantial improvements in accuracies compared to the tumor region, with ring-form partitioning further enhancing accuracies. These results highlight the feasibility and effectiveness of the proposed method for classifying brain tumors in T1-weighted CE-MRI scans. Deepak et al. (2021) [23] automated tumor characterization is crucial for computer-aided diagnosis (CAD) systems, especially in identifying brain tumors using MRI scans. However, the limited availability of large-scale medical image databases limits the training data for deep neural networks. A proposed solution is combining convolutional neural network (CNN) features with support vector machine (SVM) for medical image classification. The fully automated system, evaluated using the Figshare open dataset, achieved an overall classification accuracy of 95.82%, surpassing state-of-the-art methods. Experiments on additional brain MRI datasets validated the enhanced performance, with the SVM classifier showing superior performance in scenarios with limited training data. Fatih Demir et al. (2022) [24] brain tumors pose a global threat, and Magnetic Resonance Imaging (MRI) is a widely used diagnostic tool. This study presents an innovative deep learning approach for automated brain tumor detection using MRI images. Deep features are extracted through convolutional layers, and a new multi-level feature selection algorithm called L1NSR is applied. Superior classification performance is achieved using the Support Vector Machine (SVM) algorithm with a Gaussian kernel. The methodology achieves 98.8% and 96.6% classification accuracies, respectively. Navid Ghassemi et al. (2020) [25] this paper presents a deep learning method for classifying tumors in MR images. The method starts with pre-training a deep neural network using diverse datasets. The network then fine-tunes to distinguish between three tumor classes using six layers and 1.7 million weight parameters. Techniques like data augmentation and dropout are used to mitigate overfitting. The method outperforms state-of-the-art techniques in 5-fold cross-validation. Shahriar Hossain et al. (2023) [26] this study focuses on multiclass classification of brain tumors using deep learning architectures like VGG16, InceptionV3, VGG19, ResNet50, InceptionResNetV2, and Xception. It proposes a transfer learning-based model, IVX16, which combines insights from top three models. Experimentation yields peak accuracies of 95.11%, 93.88%, 94.19%, 93.88%, 93.58%, 94.5%, and 96.94% for VGG16, InceptionV3, VGG19, ResNet50, InceptionResNetV2, Xception, and IVX16. Explainable AI is used to assess model performance and reliability. Lokesh Kumar et al. (2021) [27] the increasing number of brain tumor cases necessitates the development of automated detection and diagnosis methods. Deep neural networks are being explored for multi-tumor brain image classification. However, these networks face challenges like vanishing gradient problems and overfitting. A deep network model using ResNet-50 and global average pooling is proposed, which outperforms existing models in classification accuracy, with mean accuracies of 97.08% and 97.48%, respectively. Nirmalapriya et al. (2023) [28] brain tumors pose a significant health risk, and manual classification is complicated by MRI data. An innovative optimization-driven model is proposed for classifying brain tumors using a hybrid segmentation approach. This model merges U-Net and Channel-wise Feature Pyramid Network for Medicine (CFPNet-M) models, using Tanimoto similarity. The model accurately segments and classifies both benign and malignant tumor samples. The SqueezeNet model is trained into four grades, and the model weights are optimized using Fractional Aquila Spider Monkey Optimization (FASMO). The model achieves 92.2% testing accuracy, 94.3% sensitivity, 90.8% specificity, and 0.089 prediction error.

The proposed ResNet101 coupled with CWAM (Channel-wise Attention Mechanism) aims to address the demerits and research gaps identified in previous studies regarding brain tumor classification using MRI data. These include challenges such as limited classification accuracy, overfitting, and the need for more effective feature extraction methods. ResNet101, known for its strong performance in image classification tasks, serves as the backbone network to extract high-level features from MRI images with greater accuracy, thus improving classification performance. Additionally, the CWAM technique helps mitigate overfitting by selectively attending to informative channels in the feature maps, reducing noise and enhancing the model’s ability to generalize to new data. By focusing on relevant channels in the feature maps, CWAM enhances the feature extraction process, enabling the model to capture more meaningful information from MRI images and leading to improved classification accuracy. Table 1 illustrates the addressed various limitation of the state-of-the-art methods.

**Table 1:** Limitation of state-of-the-art-methods

Author	Year	Dataset	Method	Limitation
[16]	2019	BRATS	CNN	The text underscores the CNN model’s success in brain tumor classification from MRI data but overlooks potential limitations or challenges, indicating the importance of comprehensive research.
[17]	2024	FigShare, Kaggle	ResNet101, DenseNet121, and EfficientNetB0	The proposed method’s limitations include its reliance on pre-trained models, which may not capture all unique features of brain tumor images, potentially limiting its adaptability and flexibility.
[18]	2022	Kaggle	CNN	The proposed deep-learning framework, while achieving high accuracy on the same machine, may not be robust enough to handle MRI images from different machines or protocols.
[19]	2022	BreakHis	ShuffleNet, Inception-V3Net	The proposed method’s limitations include its reliance on the BreakHis dataset, which may introduce bias and limit its generalizability beyond the BreakHis dataset.
[20]	2023	Kaggle	DCNN	The summary critiques the proposed approach for brain tumor classification due to its lack of specificity, suggesting its clinical applicability may be limited.
[21]	2020	HER, PACS	CAD, DCNN	Computer-aided diagnosis (CAD) systems, while promising for early-stage breast cancer detection, may increase recall rate and reading time without proper validation, requiring rigorous training and understanding.
[22]	2015	CE-MRI	GLCM, BoW	The study’s limitations include its limited exploration of augmentation techniques and partition schemes, and its exclusive focus on T1-weighted CE-MRI brain tumors, highlighting the need for further investigation.
[23]	2021	FigShare	CNN	The study’s limitations include limited medical image databases, limiting the generalizability of the CNN-SVM classification approach, and necessitating further research on larger datasets.
[24]	2022	FigShare	RCNN	The study highlights the importance of understanding false positives and negatives in classification results, despite high accuracies, to assess the model’s practical utility and suitability for clinical applications.

## Materials and methods

Deep learning models play a vital role in classifying brain scans, detecting intricate patterns for accurate diagnosis. Integrating the ResNet101-CWAM fusion technique further enhances diagnostic precision by capturing nuanced brain image features. This methodology enriches the model’s understanding of brain conditions, ensuring accurate detection and classification. The process involves meticulous data gathering, preprocessing, model selection, and rigorous training and testing to ensure optimal functionality in real-world scenarios.

### Material and pre-processing

This study uses a dataset of 7,023 brain MR images categorized into four classes: glioma, meningioma, no tumor, and pituitary [29]. The dataset is pre-processed to ensure uniformity and compatibility, with a standardized dimension of 256 × 256 pixels for seamless integration into the model architecture. The min-max normalization technique is employed to mitigate overfitting and improve computational efficiency. The dataset is then enhanced through the Fuzzy dynamic histogram equalization (FDHE) algorithm [30], which improves contrast and overall quality of medical images. This algorithm enhances the visual fidelity of brain MR images, improving the effectiveness and reliability of subsequent analysis and classification tasks. The dataset preparation process involves a series of steps to optimize the dataset’s utility and maximize the model’s performance in accurately classifying brain conditions from MR images.

**Table 2:** Dataset image split-up description

Grade	Train (70%)	Test (30%)	Total
Benign	1407	603	2010
Glioma	1135	487	1622
Meningioma	1153	493	1646
Pituitary	1230	526	1756

**Fig. 1:**
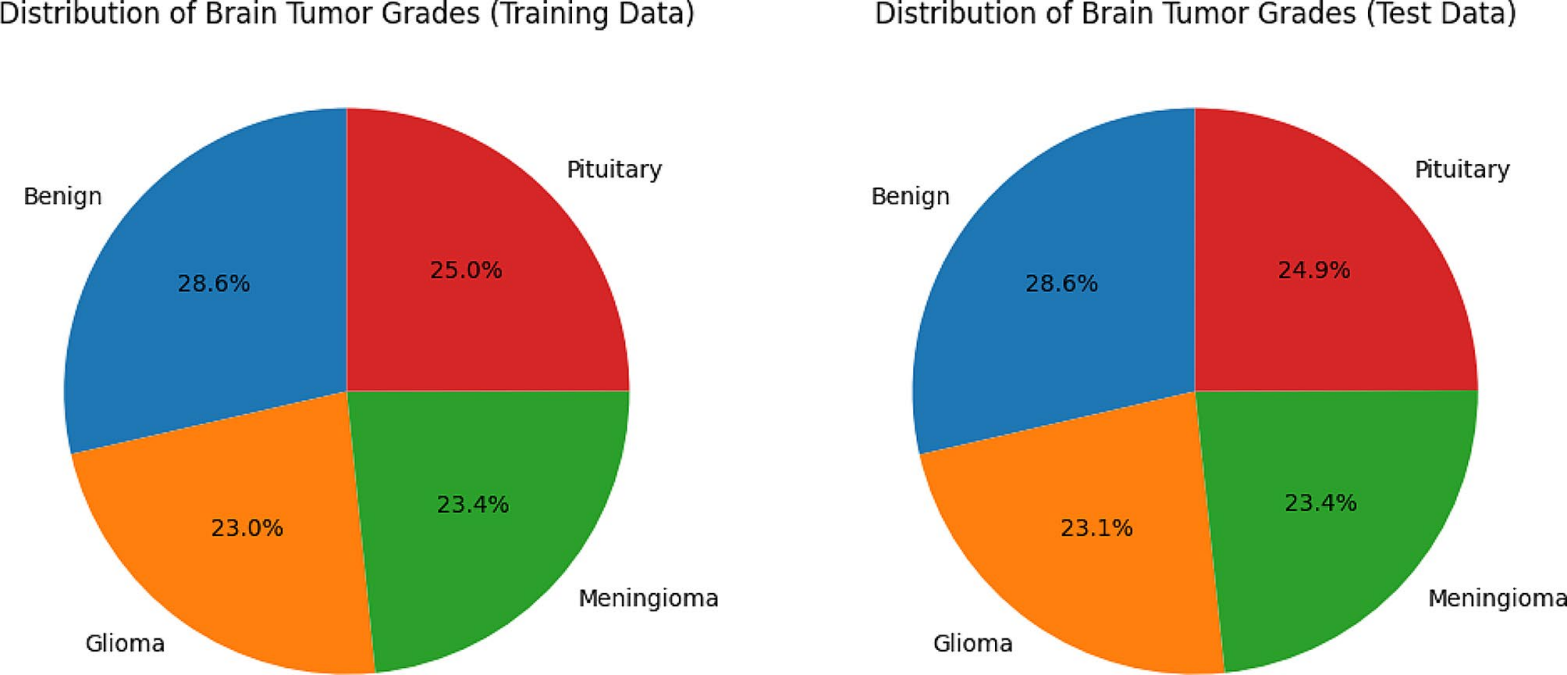
Distribution of dataset for training and testing

The FDHE algorithm contributes to the overall effectiveness and reliability of subsequent analysis and classification tasks. Table 2 illustrates dataset summary. Figure 1 depicts the dataset distribution towards the training and testing phase. The dataset, as detailed in the table, exhibits a breakdown of the brain MR images across different grades, distinguishing between benign tumors, gliomas, meningiomas, and pituitary tumors. Within the training set, which constitutes 70% of the total dataset, there are 1407 benign images, 1135 glioma images, 1153 meningioma images, and 1230 pituitary images. On the other hand, the test set, comprising 30% of the total dataset, contains 603 benign images, 487 glioma images, 493 meningioma images, and 526 pituitary images. Summing up the training and test sets, the dataset totals 2010 benign images, 1622 glioma images, 1646 meningioma images, and 1756 pituitary images. This detailed breakdown provides valuable insights into the distribution of images across different tumor types, facilitating effective training and evaluation of the deep learning model on a diverse range of data samples.

### Proposed method

**Fig. 2:**
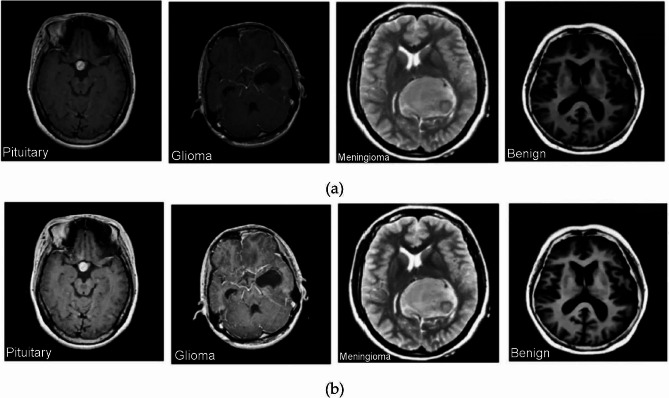
Representation of images (**a**) prior FDHE, (**b**) post FDHE

Deep learning models are crucial for classifying brain scans into various tumor types. These models can detect intricate patterns in raw image data, enabling high accuracy and efficiency in diagnosis and treatment planning. To further refine diagnostic precision, the ResNet101-CWAM fusion technique is integrated, focusing on capturing the nuances of brain images and their contextual relationships. This fusion methodology enriches the model’s understanding of various brain conditions, enhancing its ability to accurately detect and classify them. The process involves meticulous data gathering, preprocessing, model selection, and rigorous training and testing. Data is assembled to ensure representative samples, and preprocessing refines and standardizes the collected data for training. Model selection involves careful consideration of various architectures and techniques, and the model undergoes rigorous testing to ensure optimal functionality and reliability in real-world scenarios. Good contrast is essential for clear and impactful visual content, making it easier to understand messages. Techniques like FDHE help improve contrast by adjusting overly bright or dark images, making details stand out more. The study focused on fixing brightness issues and making visual details clearer, making the viewing experience better. The transformation of dataset classes before and after FDHE was demonstrated in Fig. 2, demonstrating the efficacy of the technique in revitalizing visual content. To ensure optimal performance, preprocessing steps were taken, including resizing, normalization, and histogram equalization. The model was trained using a curated training set and underwent iterative refinement. After training, the model was tested using dedicated testing sets to evaluate its efficacy in accurately interpreting and analyzing the visual data. This systematic approach showcases the transformative power of contrast enhancement techniques and underscores their pivotal role in unlocking the true potential of visual content, enabling it to be scrutinized and interpreted with precision and clarity.

The procedure involves breaking down a low-contrast image into sub-histograms based on its median value, using a histogram-based methodology. This involves meticulous examination of every pixel within the image and delineating clusters based on prominent peaks. This process persists until no additional clusters appear, indicating completion. Histogram-based equalization has an inherent advantage as it requires only a singular pass for each individual pixel. Dynamic Histogram Equalization (DHE) starts by smoothing each histogram, then identifies local maxima points by comparing histogram values with neighboring pixels. The algorithm calculates the histogram’s length, ensuring a balanced enhancement distance. The novelty of the approach lies in the integration of the Channel-wise Attention Mechanism (CWAM) with the ResNet101 architecture for the classification of MRI brain images, which represents a significant innovation in the field of medical image analysis. This combination enhances the model’s ability to focus on pertinent features within the images, thereby improving classification accuracy for various brain tumor types, including glioma, meningioma, no tumor, and pituitary classes. Furthermore, the study’s meticulous data preprocessing techniques ensure high-quality input for training the deep learning model, contributing to its impressive performance. By proposing this advanced method and advocating for its integration into clinical decision-making software, the research not only demonstrates immediate practical applicability but also sets the stage for future advancements through the identification of computational complexities and suggestions for further research.

#### Smoothing

Noise infiltrates high-frequency elements of an image, introducing jagged artifacts that can disrupt the viewing experience and obscure important details. To counteract these effects, a smoothing technique is employed by adjusting the intensity levels of individual pixels, preserving crucial details while reducing the prominence of noise-induced artifacts. The Gaussian function is central to this process, which dynamically alters the intensity of pixels to achieve a more uniform and visually appealing result [31]. Each pixel undergoes a transformation targeting the removal of blur, a common consequence of noise interference. This transformation adheres to the principles of the normal distribution, ensuring adjustments are statistically coherent and consistent with natural visual perception. Applying this transformation to every pixel enhances the overall clarity and fidelity of the image, resulting in a more visually pleasing and informative representation.

1 $$X\left(a,b\right)=\frac{1}{2\pi {\sigma }^{2}}{e}^{\frac{-{a}^{2}+{b}^{2}}{2{\sigma }^{2}}}$$

Here, ‘a’ represents the distance between the origins of the horizontal axes, ‘b’ denotes the distance between the origins of the vertical axes, and ‘σ’ signifies the standard deviation. Consequently, the smoothed image gains flexibility for Contrast Enhancement (CE). This function effectively eliminates redundant, minimal, and maximal noisy peaks, thereby enhancing the image’s quality. Following this smoothing process, the maximum points on the Receiver Operating Characteristic (ROC) curve are identified, facilitating the separation of the darkest and brightest points within the region.

#### Finding local maxima

Local maxima in a histogram are points where the intensity value peaks above its neighboring values, indicating significant features in the image. They serve as reference points for identifying the darkest and brightest areas [32]. To locate these local maxima and minima, the histogram of the smoothed image is analyzed, tracing the highest and lowest intensity values. Intensity 0 represents the lowest, and 255 the highest. Partitioning the image based on these extreme values divides it into segments. This segmentation relies on histograms to define boundaries between regions, using a histogram-based method for accuracy. In this context, the median is determined from the image histogram. The median is computed by,

2 $${K}_{meidan}={I}_{m}+\left[\frac{\frac{N}{2}{E}_{m-1}}{{e}_{m}}\right]B$$

where, ${I}_{m}$ is the lowest value of median, N is the number of observations, ${E}_{m-1}$ is a Cumulative frequency, ${e}_{m}$ is the frequency of each image and B is a median value. The image is divided into segments using this median value. The intervals between successive local maxima are termed as intervals. Partitioning is necessary to group related pixel values together, facilitating ease of analysis.

#### Proposed resNet101-CWAM approach

**Fig. 3:**
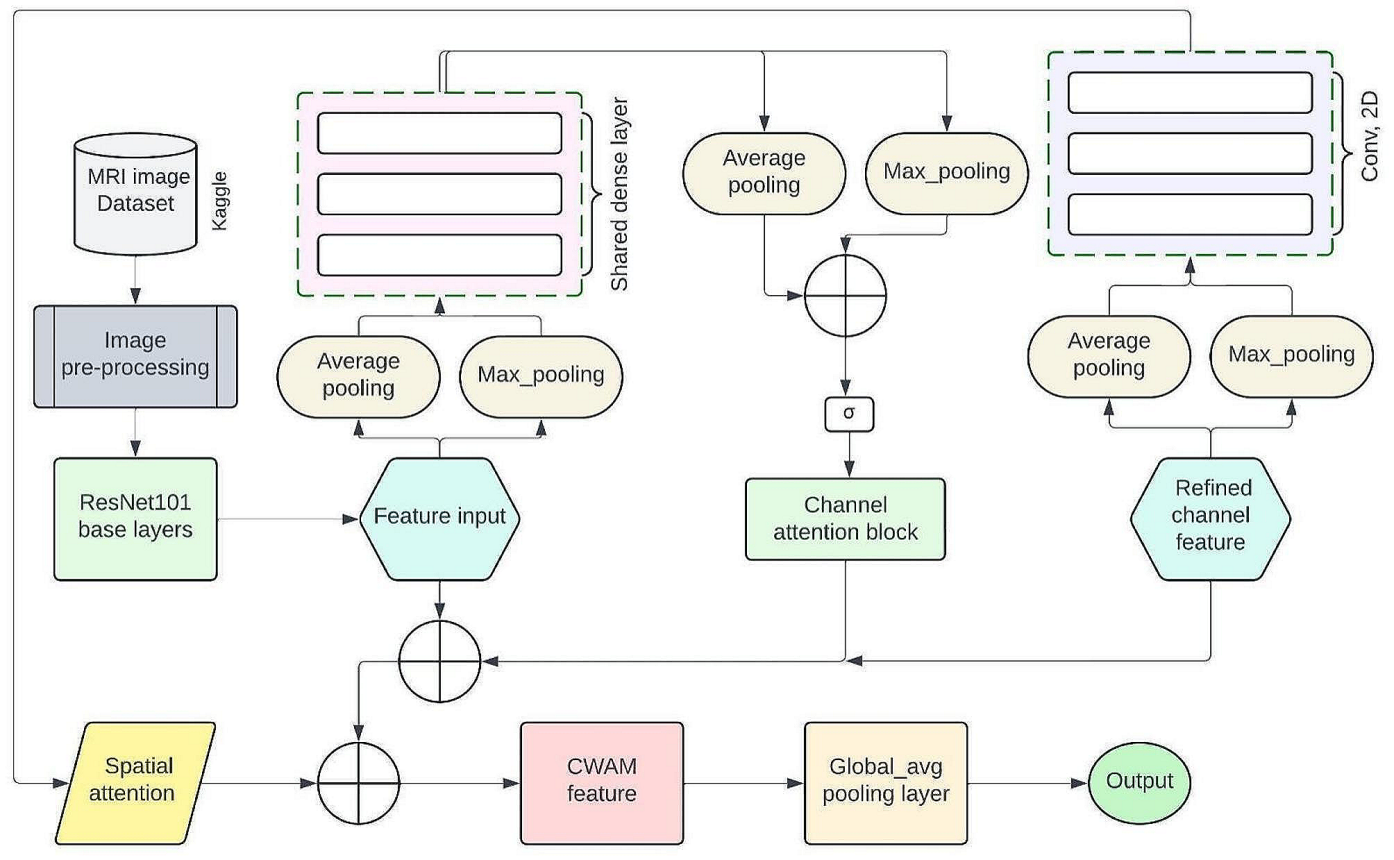
Proposed model detailed architecture

In this study, we utilized ResNet101 as our primary model architecture, leveraging pre-trained weights from the ImageNet dataset. This allowed for the extraction of intricate features from our meticulously pre-processed images, establishing a strong foundation for subsequent analysis. To maintain model stability, we froze the weights of convolutional and max-pooling layers during training, ensuring the preservation of valuable knowledge [33]. ResNet was chosen for its exceptional performance across various computer vision tasks and its ability to address the vanishing gradient problem. By harnessing ResNet’s strengths and pre-trained weights from ImageNet, we aimed to equip our model with the capabilities necessary for effective task handling, ultimately striving for optimal performance and insightful outcomes. Features from ResNet101 were extracted and input into CWAM, a framework integrating spatial and channel-wise attention mechanisms [34]. Channel attention evaluates individual channel importance by adjusting weights, enhancing the model’s focus on significant features. Spatial attention directs focus to specific spatial locations, enabling detailed analysis. Despite their distinct roles, these mechanisms synergize, maximizing the model’s ability to extract relevant information from data. CWAM’s collaborative approach ensures nuanced pattern recognition, leading to accurate insights. Figure 3 depicts the detailed architecture of the brain tumor classification model.

The feature extraction process uses ResNet101 architecture’s layers to generate a feature map with dimensions C representing the number of channels and H and W representing the spatial dimensions. This map provides a comprehensive understanding of the spatial structure and content encoded within the extracted features, highlighting the richness of information captured within each channel. The Channel-wise Attention Module (CWAM) integrates spatial and channel-wise attention mechanisms to enhance feature refinement. The input feature map undergoes transformations such as max pooling and average pooling layers to condense spatial dimensionality. The global average pooling layer computes the mean value for each channel across spatial dimensions, while the global max pooling layer identifies maximum values per channel. The Channel Attention Map (CAM) is computed through dense layers to reveal the significance of each channel, facilitating channel-specific refinement. The CAM is then multiplied element-wise with the original feature map F, resulting in a refined feature map denoted as R. Each element in R is weighted according to its channel’s importance, enhancing the discriminative power of the features for subsequent stages of analysis. Table 3 demonstrates the building block for proposed ResNet101 model.

**Table 3:** Building blocks of proposed reseNet101 architecture

Layer	Output	101-layers
conv1	112 × 112	7 × 7, 64, stride 2
conv2_x	56 × 56	3 × 3 max pool, stride 2
		$$\left[ {\begin{array}{*{20}{c}}{1 \times 1,}&{64} \\ {3 \times 3,}&{64} \\ {1 \times 1,}&{256} \end{array}} \right] \times 3$$
conv3_x	28 × 28	$$\left[ {\begin{array}{*{20}{c}}{1 \times 1,}&{128} \\ {3 \times 3,}&{256} \\ {1 \times 1,}&{512} \end{array}} \right] \times 4$$
conv4_x	14 × 14	$$\left[ {\begin{array}{*{20}{c}}{1 \times 1,}&{256} \\ {3 \times 3,}&{256} \\ {1 \times 1,}&{1024} \end{array}} \right] \times 23$$
conv5_x	7 × 7	$$\left[ {\begin{array}{*{20}{c}}{1 \times 1,}&{512} \\ {3 \times 3,}&{512} \\ {1 \times 1,}&{2048} \end{array}} \right] \times 3$$
	1 × 1	3 × 3 average pool, 1000-d fully connected

The model employs a meticulously crafted feature map to delve into the essence of crucial features residing within each channel. At the heart of this pursuit lies the spatial attention module, which orchestrates the compression of the channel-refined feature map through operations such as maximum and average pooling. This transformation results in two distinct 2D representations, each providing insights into the spatial intricacies ingrained within the data. Within this framework, the attention map serves as a conduit between spatial and channel-wise dimensions. Integrated seamlessly with the channel-refined feature map R, this amalgamation provides a nuanced understanding of both spatial context and channel-specific significance, enriching the model’s comprehension of the data landscape. As the journey progresses, the CWAM module emerges as a cohesive force, merging spatial and channel-wise attention to refine features comprehensively. This amalgamated output encapsulates the core of feature refinement, ready to reveal hidden truths within the data. Through global average pooling, the model engages in a collective contemplation of the statistical attributes of the feature space, delving deeper into the essence of the data. Finally, as the fully connected layer activates with SoftMax, the model’s insights are refined and ready for action, enabling it to navigate the intricate data terrain with confidence, extracting valuable insights and informing strategic decisions.

#### Performance metric parameters

The evaluation of the performance of the suggested model has been completely comprehensive, taking into account a wide range of important characteristics to determine how successful it is. A few examples of these parameters are as follows: accuracy (Acc), which is a measurement of the proportion of instances that have been correctly classified out of the total number of instances; precision (Pr), which evaluates the accuracy of positive predictions; F1-score, which is a harmonic mean of precision and recall that provides a balanced assessment of the model’s performance; and recall, which evaluates the proportion of true positive instances that were correctly identified by the model. By taking into account these many characteristics, we are able to get a full picture of the capabilities and limits of the model in relation to various elements of classification accuracy and prediction performance.

3 $$Acc=\frac{T.positive+T.negative}{T.positive+T.negative+F.positive+F.negative}$$

4 $$Pr= \frac{T.positive}{T.positive+F.positive}$$

5 $$Recall=\frac{T.positive}{T.positive+F.negative}$$

6 $$F1 score=\frac{2*pr*recall}{pr+recall}$$

## Experimental results and discussion

To ensure robustness and reliability in the performance assessment of the ResNet101-CWAM model, a rigorous approach was used throughout the training and evaluation phases of the performed research. The key component of this strategy was the use of a five-fold cross-validation methodology, which is a well-known machine learning technique for reducing bias and variance problems related to model training and assessment. The dataset was first divided into two parts: 30% of the data was put aside for validation and 70% of the data was used for training. This partitioning strategy was developed to provide for a thorough evaluation of the model’s generalizability by keeping a distinct set for independent validation and supplying the model with enough data for learning. As a crucial litmus test for assessing the model’s efficacy outside of the training data, the testing dataset was also kept outside from the training and validation sets. Because of this division, the model’s performance was examined on hypothetical data, yielding insightful information about how applicable it would be in practice. In order to do a rigorous analysis of the model’s durability and adaptability, the dataset was carefully split into five sets, which each functioned as a separate fold during the cross-validation technique. These sets were then subjected to iterative cycles of training and validation, enabling a thorough investigation of the model’s behaviour over a range of data configurations. A range of performance parameters, including as accuracy, precision, recall, and the area under the receiver operating characteristic (ROC) curve (AUC), were used to evaluate the model’s performance. These measures offered complex insights into many facets of the model’s predictive power, facilitating a more nuanced comprehension of its advantages and disadvantages. The study ensured a fair and accurate evaluation of the ResNet101-CWAM model’s performance through a comprehensive evaluation method. This approach provided valuable insights into the model’s strengths and weaknesses, contributing to both scientific rigor and our knowledge of computational biology and machine learning.

**Table 4:** Hyperparameters in the ResNet101-CWAM model

Parameters	Model-I	Model-II
Rate of learning	0.001	0.001
Size of batches	32	16
Optimizing method	Adam	SGD
No. of epochs	25	25

In the function of an extensive library, Table 4 explains the intricate hyperparameters that are carefully defined within the network architectural design. In the process of looking for optimisation, several different types of optimizers were carefully examined. As the table illustrates, Adam and Stochastic Gradient Descent (SGD) emerged as significant rivals among these optimizers. Model-I superior adaptive learning rate mechanism had a role in the decision to choose Adam as the optimizer. This dynamic characteristic enables the model to adapt to nonstationary gradients and navigate complex loss landscapes with success. Adam’s flexibility allows him to quickly converge and become more broadly oriented, making him particularly good at overcoming the many challenges that come with complex tasks. However, there were additional practical considerations that led to the decision to use SGD as the optimizer for Model-II. The design of Model-II benefited from SGD’s inherent simplicity and demonstrated performance across a variety of domains, since it satisfied the exact requirements and architectural constraints. Moreover, SGD’s resource-efficient feature aligns well with the computational constraints encountered in real-world deployment scenarios, making it a logical choice for maximising model performance.

In conclusion, a sophisticated approach to hyperparameter tuning is highlighted by the deliberate selection of optimizers that are appropriate for the unique qualities and demands of each model. The goal of this approach is to maximise effectiveness and performance in a variety of settings and applications. The table provides a comprehensive overview of the key hyperparameters configured for Model I and Model II. In Model I, the learning rate was set at 0.001, enabling the model to adjust its weights gradually during training to minimize the loss function. The batch size for Model I was determined to be 32, indicating that 32 samples were processed simultaneously before updating the model’s parameters. Adam was selected as the optimizing method for Model I, leveraging its adaptive learning rate feature to navigate complex loss landscapes effectively. The number of epochs for Model I was established at 25, signifying the number of times the entire dataset was passed forward and backward through the neural network during training. Conversely, Model II maintained a similar learning rate of 0.001 but opted for a smaller batch size of 16, potentially enhancing the model’s sensitivity to subtle patterns within the data. SGD was chosen as the optimizing method for Model II due to its simplicity, resource efficiency, and proven effectiveness in numerous applications. Like Model I, Model II was trained for 25 epochs, ensuring thorough exploration of the dataset while mitigating the risk of overfitting.

**Table 5:** Performance metric evaluation of the proposed ResNet101-CWAM

Dataset split	Training test			Five-fold cross validation test			
				Model-I		Model-II	
Performance metric	Model-I (%)	Model-II (%)	Avg (%)	Mean (%)	Std_deviation (%)	Mean (%)	Std_deviation (%)
F1-score	99.27	97.08	98.175	98.82	0.02	97.88	0.013
Re-call	99.21	97.11	98.16	98.83	0.024	97.12	0.022
Accuracy	99.83	98.77	99.30	99.41	0.008	98.98	0.013
Precision	99.06	98.05	98.555	99.02	0.015	98.06	0.016
AUC	99.33	98.13	98.73	99.12	0.014	97.95	0.014

**Fig. 4:**
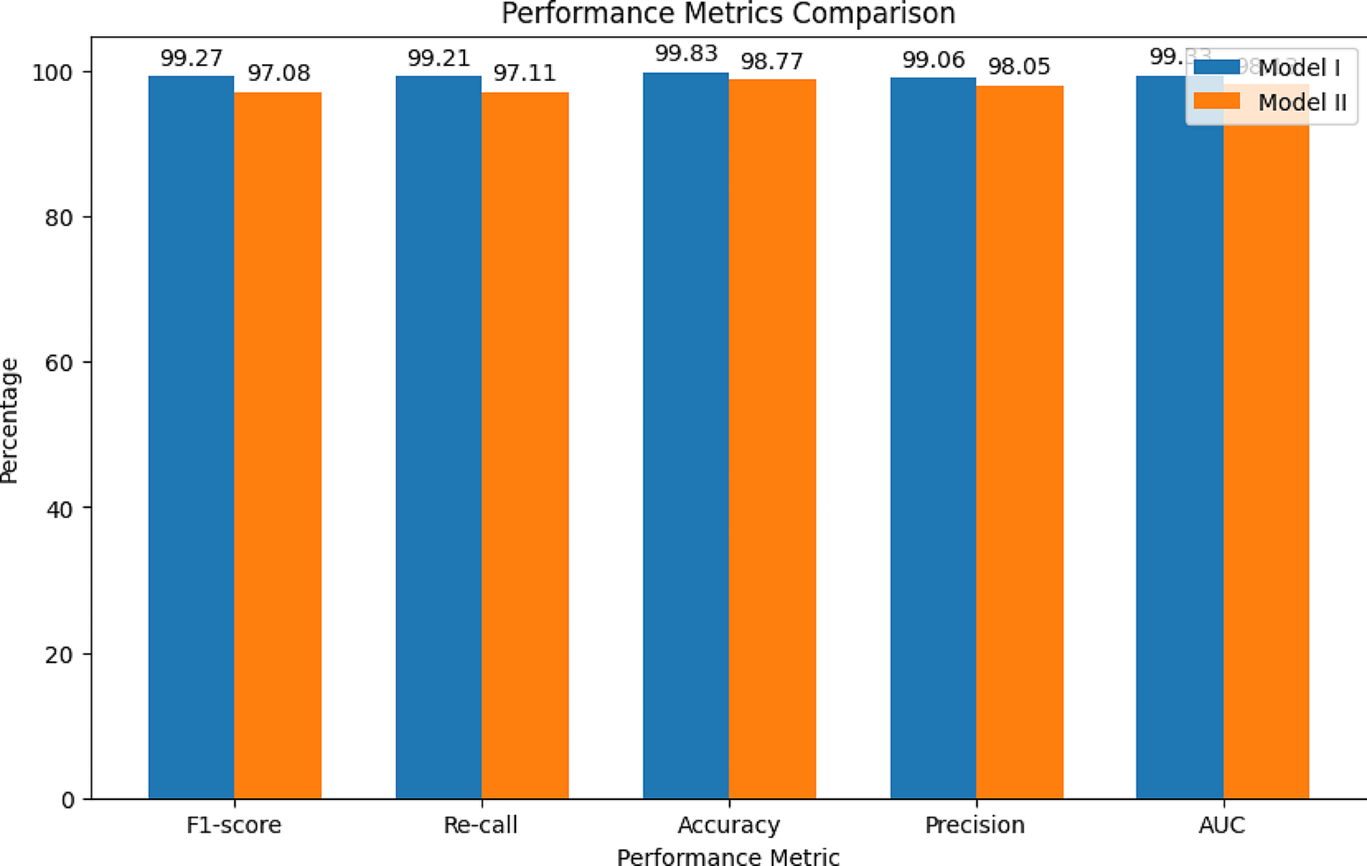
Evaluating the performance of ResNet101-CWAM for brain tumor classification

The provided Table 5 demonstrates a detailed breakdown of performance metrics for Model I and Model II across various dataset splits, encompassing both the training set and the results of five-fold cross-validation tests. For Model I, notable achievements include an impressive F1-score of 99.27%, recall of 99.21%, accuracy of 99.83%, precision of 99.06%, and AUC of 99.33% on the training dataset. During cross-validation, the model sustained high performance, with an average F1-score of 98.82%, recall of 98.83%, accuracy of 99.41%, precision of 99.02%, and AUC of 99.12%, exhibiting minimal standard deviation across these metrics. Conversely, Model II demonstrated slightly lower performance metrics on the training dataset, with an F1-score of 97.08%, recall of 97.11%, accuracy of 98.77%, precision of 98.05%, and AUC of 98.13%. Throughout cross-validation, Model II maintained consistency with an average F1-score of 97.88%, recall of 97.12%, accuracy of 98.98%, precision of 98.06%, and AUC of 97.95%, indicating a marginally higher standard deviation across these metrics compared to Model I. Figure 4 depicts the performance metric comparison of two models.

**Fig. 5:**
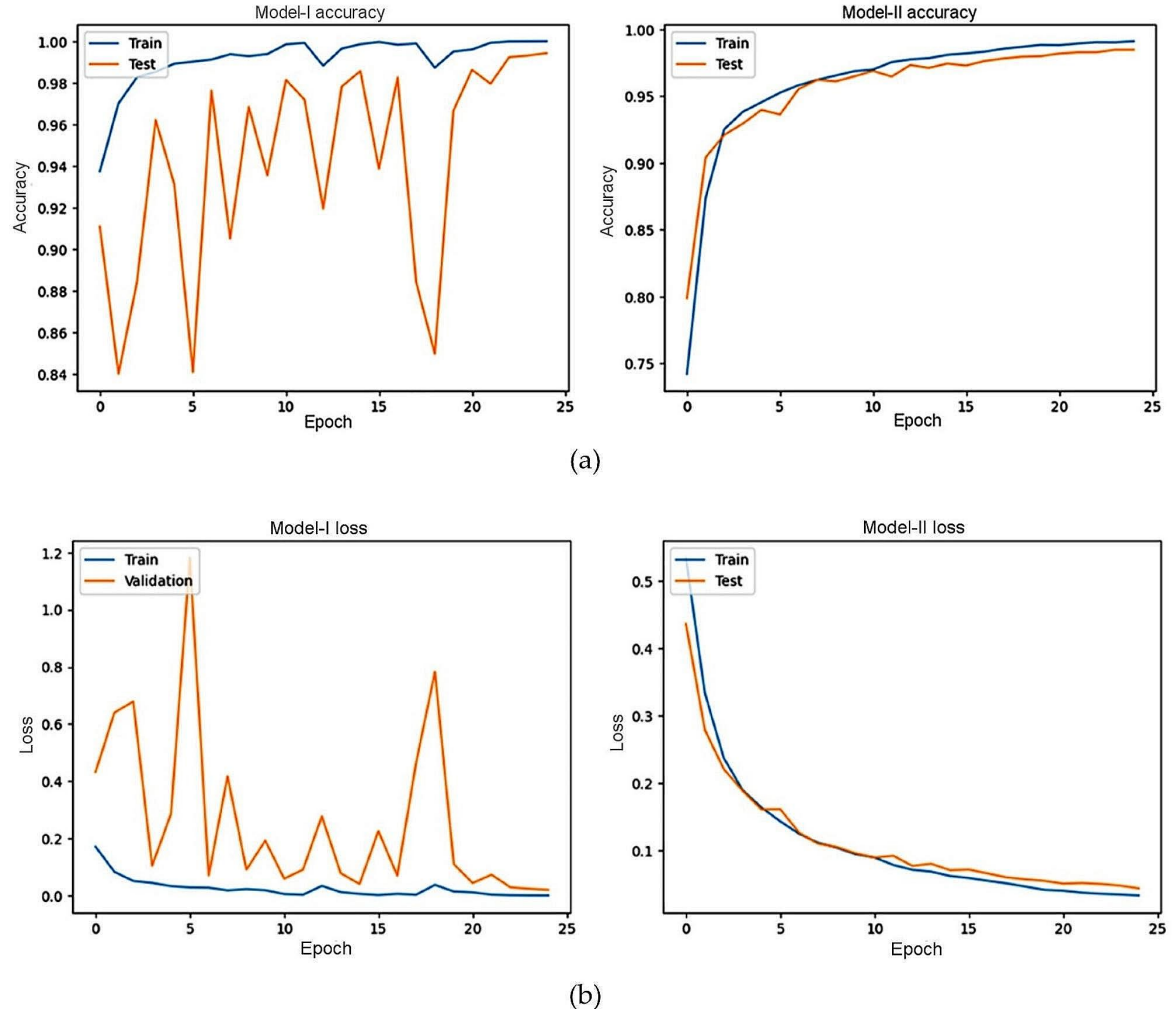
The train and test accuracy of (**a**) model-I, (**b**) model-II

The patterns that can be seen in the models’ accuracy and loss graphs correspond to the well-established characteristics of the Adam (I) and SGD (II) optimisation techniques. Not only does Adam employ adaptive learning rates to effectively navigate complex loss landscapes, but it is also highly respected for its ability to fast reach early convergence. However, since Adam’s optimisation process is dynamic, fluctuations may sometimes disrupt this rapid convergence in the early training stages. This might have something to do with Adam’s dynamic optimising process. SGD, on the other hand, often exhibits a convergence trajectory that is more gradual and is marked by modest advancement and a kinder descent towards optimal solutions. Despite these modifications, the models’ resilience and robustness may be deduced from the significant stability and consistency shown in performance metrics for both optimizers. Regardless of the optimisation method used, the models’ capacity to provide consistent performance is shown by the smallest standard deviation displayed in these metrics. Consequently, confidence in the models’ reliability and efficacy for real-world applications is reinforced. Figure 5 illustrates the training and testing accuracy and loss curves for two models.

**Fig. 6:**
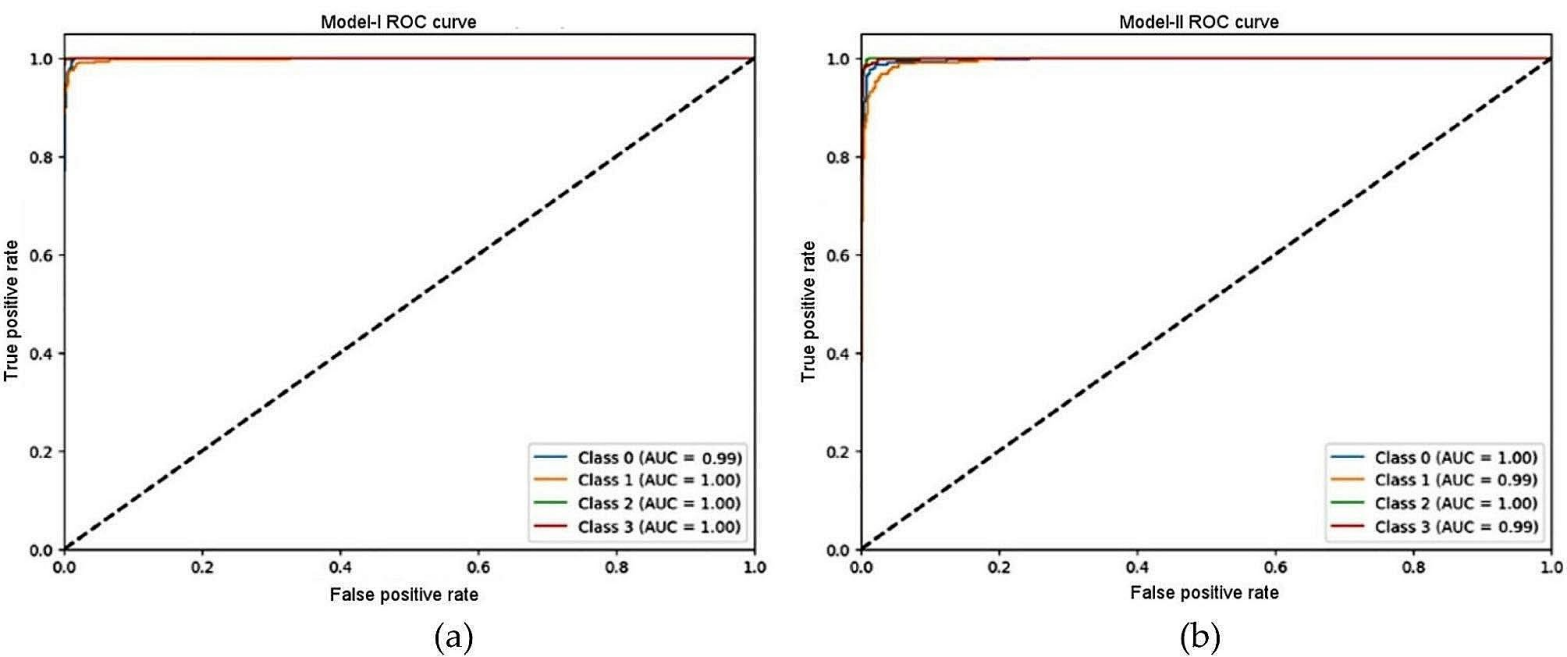
ROC curves for (**a**) model-I, (**b**) model-II

**Fig. 7:**
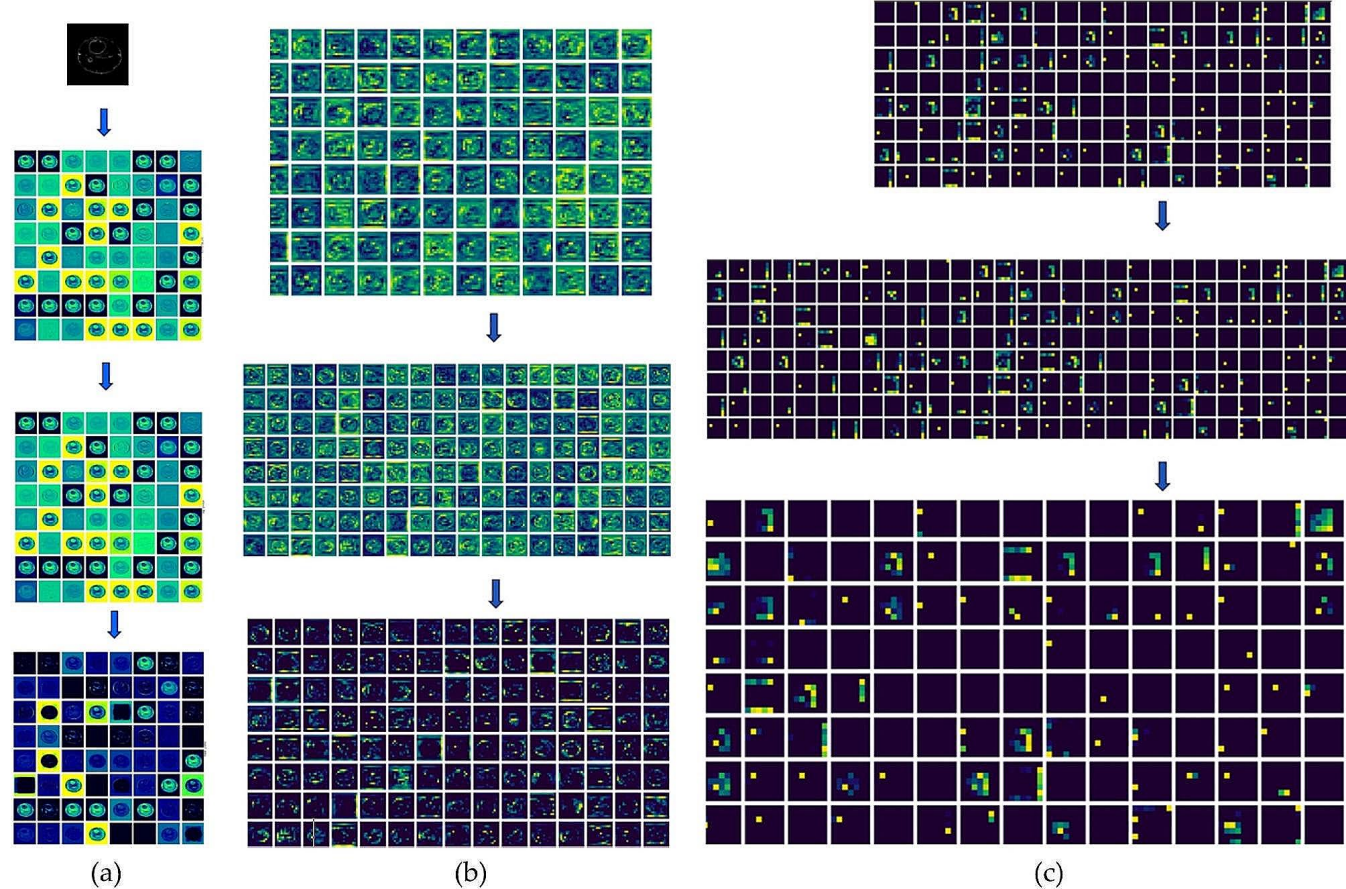
Feature maps of (**a**) first layer, (**b**) middle layer, (**c**) final layer

The receiver operating characteristic (ROC) curve plots are shown in detail in Fig. 6, which also offers insights into how well the models perform over a range of categorization criteria. A thorough evaluation of the models’ discriminatory capacity is made possible by the way each curve illustrates the trade-off between the true positive rate (sensitivity) and the false positive rate (1 - specificity). Additionally, the models’ classification performance is quantified by the accompanying area under the curve (AUC) score for each class, which provides a detailed knowledge of the models’ capacity to discriminate between various classes. This thorough visualisation makes it easier to make decisions about how well the models work for certain categorization tasks, which improves the assessment findings’ interpretability and usefulness. We have conducted a meticulous process of visualising the feature maps, shown in Fig. 7 (a) – (c), to assess the models’ ability to comprehend the primary visual attributes of the images and the contextual relationships among them. The model consists of three levels: the beginning, intermediate, and final layers. These layers are visually represented by feature maps employed in the model.

After doing a thorough analysis of the feature maps obtained from the first three layers, it becomes evident that they possess the capability to accurately capture fundamental characteristics such as edges, textures, and basic shapes. Furthermore, this capability emphasises the crucial role that these layers have in identifying underlying patterns in the incoming data, thereby creating a foundation for further hierarchical processing within the neural network’s architecture. Looking at Fig. 7 (b) and (c), we can see that the feature maps get more abstract as the model goes deeper. This indicates their ability to capture more intricate features within brain MRIs. Figure 7 (b) is important because it shows how the CWAM module highlights specific parts in the feature maps. This shows us where important stuff is in terms of space and channels. We hope this helps make the important areas and channels clearer, which should make predictions more accurate. Simultaneously, less significant aspects of the data may not stand out as prominently. This prioritization enables us to concentrate on the critical details essential for sorting and analyzing the data effectively. Our method was meticulously compared with top-performing techniques in the field, all utilizing the same dataset. This comparative analysis was conducted due to the exceptional performance of our approach. Our ResNet101-CWAM model did better than the others, as we found out from this comparison. The details of this comparison are shown in Table 6, which helps us understand how well different methods work. It’s important to mention that we used the same training and testing methods from previous studies to test our ResNet101-CWAM model, as explained in Table 6. This ensures fairness and makes it easier to compare the different methods, which makes our results more believable and trustworthy.

**Table 6:** Performance metric comparison of proposed and other state-of-the-art methods

Authors	Models	Performance metric parameters			
		Accuracy (%)	Precision (%)	Recall (%)	F1-score (%)
Remzan et al.	EfficientNetB1, ResNet50	95.98	95.98	96.03	95.98
Tahiry K et al.	Hybrid CNN	95.65	95.65	95.67	95.65
Zhang Z et al.	VGG16	94.55	96.5	96.01	96.02
Dewan JH et al.	VGG19	97.02	96.10	97.01	97.11
Sheng M et al.	CNN	98.40	97.17	96.75	96.75
Proposed model	ResNet101 + CWAM	99.83	99.06	99.21	99.27

**Fig. 8:**
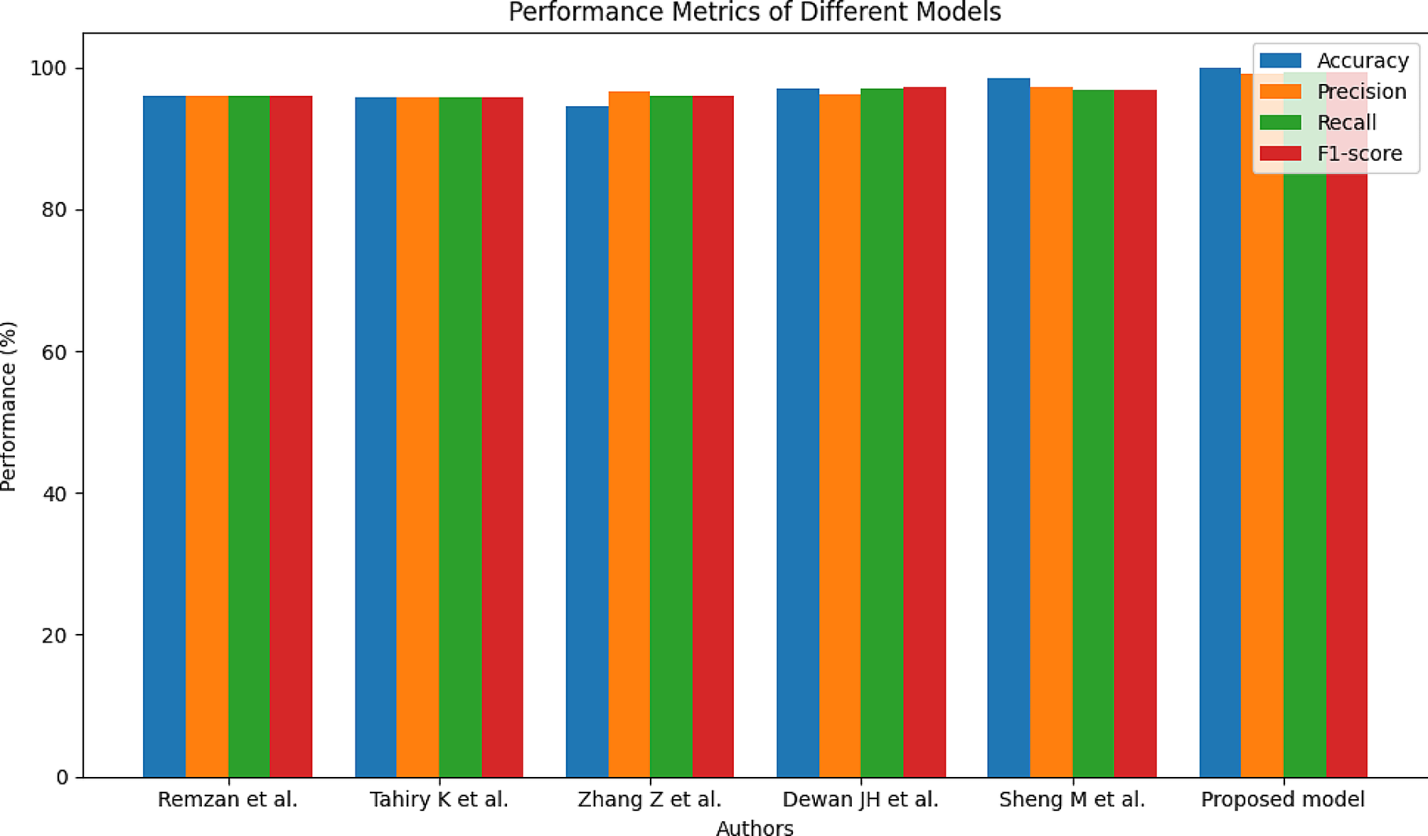
Performance metric outcome comparison of proposed and other existing models

In Table 6, evaluating various models’ performance, several authors contributed their findings. Remzan et al. employed EfficientNetB1 and ResNet50 models, achieving an accuracy of 95.98%, with corresponding precision, recall, and F1-score metrics hovering around the same high level. Tahiry K et al. introduced a Hybrid CNN model, which demonstrated commendable performance across all metrics, particularly with an accuracy of 95.65% and consistent precision, recall, and F1-score values. Zhang Z et al. explored the VGG16 model, achieving a slightly lower accuracy of 94.55% but with a higher precision score of 96.5%. Dewan JH et al. presented results from their VGG19 model, boasting an accuracy of 97.02% and notably high F1-score of 97.11%. Sheng M et al. introduced a CNN model with impressive accuracy at 98.40% and precision at 97.17%. Lastly, the proposed model, ResNet101 + CWAM, exhibited exceptional performance, achieving the highest accuracy of 99.83% and F1-score of 99.27%, indicating its robustness in classification tasks. Figure 8 represents the performance metric parameter outcome of proposed and state-of-the-art methods.

### Ablation study

Furthermore, researchers carried out a study on the model, using specific settings for how it works and dividing the data into parts, with 70% used for training the model and 30% for testing it. They put together all the findings in Table 7. In the preprocessing stage, crucial steps optimized the model’s performance. Initially, resizing images to 256 × 256 pixels ensured uniformity and compatibility, easing input. Min-max normalization prevented overfitting by scaling pixel values. Dynamic histogram equalization (DHE) further enhanced medical image quality, preserving diagnostic details. These techniques collectively bolstered the model’s performance, enabling better generalization and more reliable diagnostic outcomes. When they took out each piece of the model, it made the predictions for brain tumors less accurate. However, when they used all the parts together, their recommended method worked better than any other. This highlights how essential it is to include all the parts when trying to predict brain tumors accurately. Based on our research, Model-I performed better than Model-II both during data analysis and cross-validation. This suggests that Model-I was able to learn more effectively. One possible reason for this is that we used a technique called the Adam optimizer with Model-I. The Adam optimizer adjusts the learning speed for different parts of the model, which is useful for complex tasks. In contrast, Model-II used a different technique called SGD, which makes everything learn at the same speed. When dealing with brain tumors, there are many factors to consider, and some might require more careful attention.

The Adam optimizer helps by adapting the learning speed for different aspects of the brain tumor problem while training the model. To improve performance, it might be worth exploring methods such as teaching the model fewer things at once or using a different approach to training. The investigation on ablation provided valuable insights into the model’s functionality. It highlighted the effectiveness of the model’s attention processes in highlighting important features while minimizing irrelevant noise, which greatly contributes to its high performance. What’s particularly intriguing is the comparison between two types of attention mechanisms - Channel attention (CA) and Spatial attention (SA). The results showed that ResNet101 with Channel attention outperformed ResNet101 with Spatial attention. This suggests that, when dealing with brain tumor classification, focusing on specific features within the data may be more beneficial than considering spatial arrangements. This underscores the importance of carefully selecting and fine-tuning attention mechanisms based on the unique characteristics of the problem at hand. It’s important to mention that although ResNet101 didn’t achieve the highest performance in our experiments, it still outperformed some of the methods discussed in Table 6. This study focused on using the ResNet101-CWAM model to classify brain tumors in MR images, particularly aiming at multiclass classification. The results of our experiments show that our approach performs better than the current best ConvNet models in terms of accuracy. Additionally, MRI images have unique features and are captured using various techniques, which can make it challenging for pretrained models, commonly used in previous studies, to accurately capture the relevant medical properties of brain MRI images. By incorporating an attention mechanism into the CWAM module, we effectively addressed this challenge by highlighting important aspects of the images, as illustrated in Fig. 7(a)-(c), leading to improved model performance. Table 7 illustrates the ablation study of proposed brain tumor classification models.

**Table 7:** Proposed model for brain tumor classification ablation study

Models	Accuracy (%)	Precision (%)	F1-score (%)	Recall (%)
ResNet101	98.91	98.12	97.98	98.02
ResNet101 + CA	99.29	98.88	98.64	98.54
ResNet101 + SA	98.96	97.91	97.85	97.71
Proposed model	99.83	99.06	99.27	99.21

**Fig. 9:**
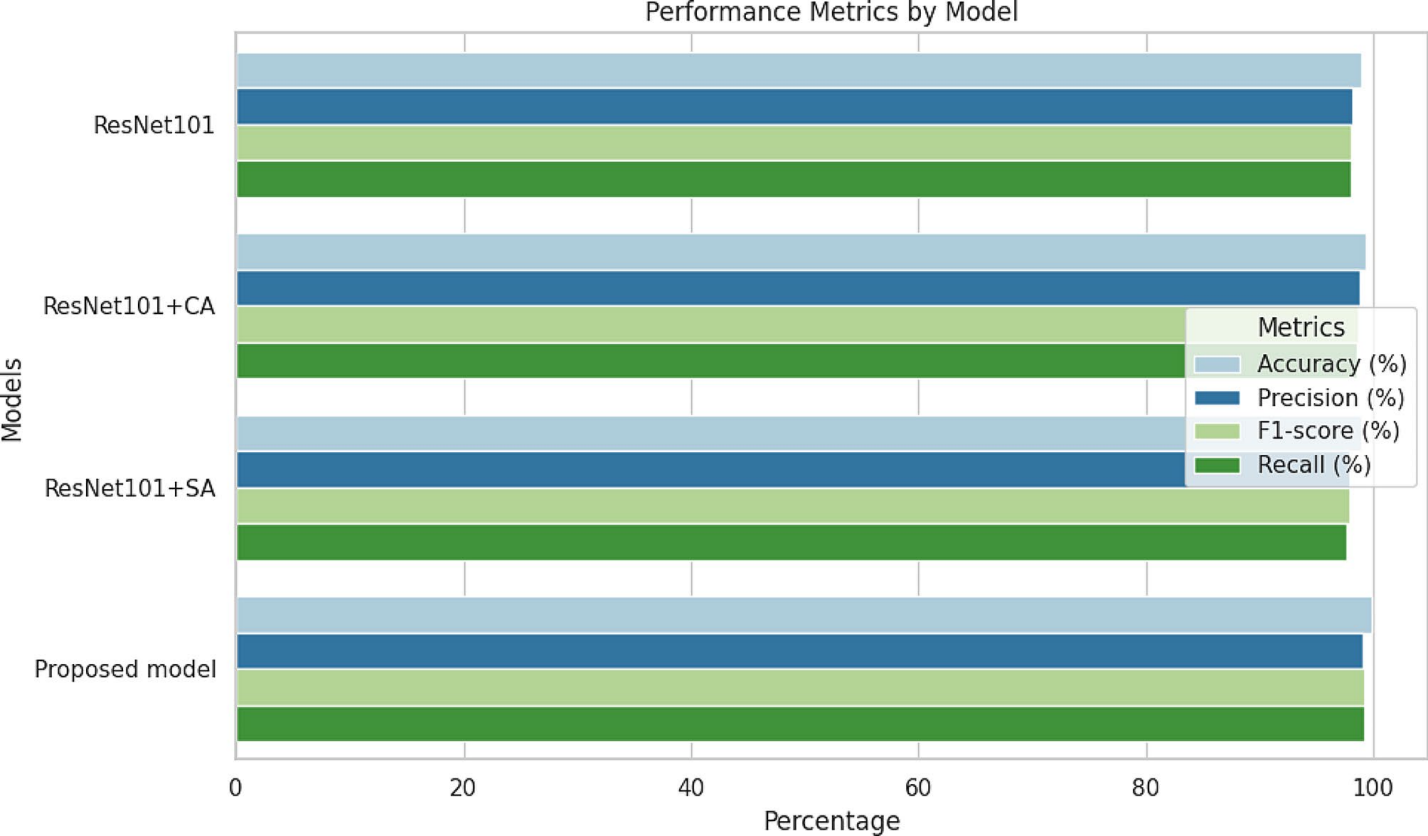
Performance metrics of models in ablation study

The Table 7 presents the performance metrics of different models in classifying brain tumors, including accuracy, precision, F1-score, and recall. The results indicate that the proposed model achieved the highest accuracy at 99.83%, with impressive precision, F1-score, and recall rates of 99.06%, 99.27%, and 99.21% respectively. This suggests that the proposed model excels in accurately identifying brain tumors with minimal false positives and negatives. Following closely behind is the ResNet101 + CA model, which demonstrates high accuracy and precision at 99.29% and 98.88%, respectively. However, the proposed model outperforms it in terms of F1-score and recall, indicating a better balance between precision and recall. The ResNet101 and ResNet101 + SA models also perform well, with accuracy rates above 98% and respectable precision, F1-score, and recall values. These findings underscore the effectiveness of the proposed model in enhancing the accuracy and reliability of brain tumor classification. Figure 9 depicts the performance metric comparison of ResNet models in ablation study.

Our research suggests that using the ResNet101-CWAM model in real clinical settings could enhance the accuracy and speed of diagnosing brain tumors. This is particularly crucial when quick identification is necessary for planning treatments and predicting patient outcomes. Healthcare providers can leverage the improved performance of the model to refine diagnostic practices and enhance overall patient care. However, when deploying such models in real clinical settings, concerns arise regarding understanding how the model makes decisions and protecting patient data confidentiality. Medical professionals need insight into the model’s decision-making process, underscoring the importance of subsequent clinical validation to ensure effectiveness, reliability, and ethical integrity. To enhance the model’s applicability across diverse patient groups and address data privacy concerns, further evaluation and the utilization of federated learning methods are vital. In future research, exploring explanation strategies that aren’t limited to one specific model, as well as considering alternative attention methods and data preparation techniques, could advance the development of brain tumor classification models. Additionally, extending this research to include 3D MRI images using volumetric attention processes could offer opportunities for more comprehensive and detailed feature extraction.

## Conclusion

In this study, we developed a deep learning-based method for accurately classifying brain tumors in medical images. Our approach effectively categorizes various types of brain MRI scans, including glioma, meningioma, no tumor, and pituitary classes. The experimental results demonstrate the outstanding effectiveness of the Channel-wise Attention mechanism framework in tumor classification, achieving an impressive accuracy of 99.83%, surpassing baseline methods. This highlights its effectiveness in precisely identifying and categorizing brain tumors. The high accuracy of our proposed technique can be attributed to the careful preprocessing of data, utilization of deep learning, and incorporation of an attention mechanism. Given the remarkable performance observed in this study, we recommend integrating our method into the software platforms used by medical professionals to enhance clinical decision-making and ultimately improve patient care. However, one limitation of our current model is its computational complexity. The incorporation of CWAM attention modules into the ResNet101 architecture introduces additional parameters and increases the model size, requiring more memory for model development. Furthermore, CWAM modules involve operations such as global pooling, convolution, and element-wise multiplication, which lead to higher computing demands. Therefore, it would be interesting for future studies to develop a more compact deep learning model integrated with attention mechanisms for brain tumor classification. In our future research, we aim to broaden the scope of our study by incorporating additional brain tumor datasets and investigating alternative deep learning approaches to enhance the accuracy of brain tumor detection further. Specifically, we plan to explore the model’s generalizability across diverse patient populations and investigate the integration of multimodal imaging data to improve diagnostic capabilities. These specific recommendations for future research directions will contribute to advancing our understanding of brain tumor detection and potentially improving patient care outcomes. Overall, in a medical setting, the ResNet101-CWAM model demonstrates the ability to effectively identify important features in brain MRI scans, enabling faster and more accurate diagnoses, improved treatment planning, and increased chances of patient survival. Moreover, reducing the likelihood of incorrect positive and negative results could alleviate patient distress.

## Data Availability

https://www.kaggle.com/datasets/masoudnickparvar/brain-tumor-mri-dataset.
